# D. D. S. or M. D.; Which?

**Published:** 1886-07

**Authors:** Geo. H. Chance

**Affiliations:** Portland, Oregon


### D. D. S. OR M. D.; WHICH?

BY GEO. H. CHANCE, D. D. S., PORTLAND, OREGON.

So much has been said and written on Dental Education that to the superficial thinker the subject may seem to have been exhausted, but as the question is still an open one, any new mode of presenting it will doubtless be acceptable to those who can and do look beneath the surface.

In previous discussions on this subject the divergence in the various views presented appears, to the writer, to have been caused mainly by a want of agreement among the disputants as to what is the true relation that dentistry of the present day sustains to the profession of general medicine.

It will be admitted, I think, that Dental Surgery is a mechanical art applied, from a medico-scientific standpoint, to the dental organs and their associate parts, for the arrest and cure of disease, precisely as general surgery is a mechanical art applied from the same standpoint to other parts of the human body, and for a similar purpose, viz.: for the arrest and cure of disease. Now, if the foregoing definition be accepted as correct, it logically follows that dental surgery is a legitimate branch of the healing art, and consequently a department of general medicine.

Having thus settled the status of our profession, it next devolves upon us to consider what is the best mode of teaching and training, and thus qualifying those who are to become dental surgeons.

If a young man desires to become a shoemaker, he certainly would not go to a tanner expecting to obtain the desired knowledge, neither would he go to a maker of lasts, or to one who makes shoe-pegs for a livelihood, simply because leather, lasts and shoe-pegs are needed in the making of a boot or a shoe. The tanner may be never so well versed in his line of business, the lastmaker may be equally competent, and the shoe-peg man may excel in the making of shoe-pegs, and each of the parties named may be able to readily distinguish between a child's shoe and a lady's slipper, or a gentleman's walking boot, but their combined knowledge could not by any stretch of the imagination constitute either or all of them competent instructors in the art of making boots and shoes.

No, the young man, if he desires to excel in the calling he has chosen, must select for his instructor one who makes the anatomy of the foot his particular study, both as to its physiological and pathological conditions, thereby enabling him to so construct a boot or a shoe that it may be made an ornament to the foot and a comfort to the wearer. Nor is this all. He must in the very nature of things (while he may not be familiar with all the details) understand the underlying principles which govern in the tanning of leather, the turning of shoe lasts and the making of shoe-pegs; this knowledge he needs to enable him to intelligently select such materials and such appliances as will meet the requirements of each particular foot. With such an instructor as is here indicated, there can be no doubt that a young man with a fair amount of constructive ability will, with proper application, in due time become successful in the calling of a shoemaker.

Let us now apply the illustration, as far as it may be applicable, to dental surgery. A young man possessing the necessary qualifications desires to enter our profession as a student. Where shall he go, and how shall he proceed to acquire the knowledge necessary to make him a first-class dentist? Please bear in mind that we speak of a dentist; not a physician or a general surgeon, but a dental surgeon—one whose life-work is to be devoted to the arrest and cure of diseases of the dental organs and their associate parts. In this connection we may ask: What are the qualifications needed before he should present himself as a student in dental surgery? It may be answered that the candidate must possess moral quality, mechanical ability and artistic taste, together with the benefit that a good common school education, including Latin, secures. He must also possess enough brain-power to enable him to mentally digest and thereby understand the principles involved in the future study and practice of the profession he has chosen.

The next question to be answered is, where shall such a young man go and how proceed?

He certainly would not go to a lawyer because the lawyer happens to be well versed in medical jurisprudence, nor to a law school where such things are taught. Neither would we expect him to go to a druggist because the druggist is a graduate from a school of pharmacy, and therefore knows all about the pharmacopœia and is an expert in compounding drugs. But let us suppose the young man has satisfied himself that dental surgery is a department of medicine, and, being ambitious as well as conscientious, thinks he ought to be an M. D. before he practices the specialty. With this thought in his mind he consults the family physician, who is an M. D. of long standing, and for whose opinions the young man's mother has a profound respect. It matters not from what or which medical school the M. D. graduated, whether it be Allopathic, Homœpathic or Eclectic, because they all graduate M. D.'s, you know, with equal rights and privileges under the law; therefore "they are all honorable men."

But this particular M. D., with all the pompous dignity so characteristic of an M. D. of that class, who assume to believe that the sum total of all medical knowledge worth having circles around and centers in those mystical awe-inspiring letters, M. D., replies to the questioner thus: "Young man, I am a physician, and therefore have neither time nor inclination to give attention to such small matters as tooth-doctoring, or what some are pleased to dignify as Dental Surgery. No, young man, I am an M. D., and my advice to you is, if you really desire to become a *professional* man, go to college and get your degree of M. D.; you can then fool away all the balance of your days on teeth if you wish. I am sure no respectable physician will raise any objections. I rather think they would commend it. In fact, I, as an old member of the medical profession, would be glad to see some of our M. D.'s take up that specialty. It would help us physicians who pay no attention to such things, and we would then have some one with whom we could consult in obscure cases of disease without lowering our dignity as professional men, for, do you know, those infernal things called teeth play the very mischief with some of our patients, so that at times we are at our wits' end to know what to do next. Several times of late that modest fellow with the absurd title of D. D. S. stuck at the end of his name, has helped me out of scrapes with my patients by telling me what was the matter; but then he knows some things about medicine and is a good, honest fellow, so you see I don't mind consulting him, especially as he saves me a great deal of trouble by attending to my children's teeth without costing me much. Yes, sir, go to college *first* and get your M. D. degree, then you can fool around teeth to your heart's content, and if you should blunder sometimes it won't make much difference; we medical men will stand by you. That's my advice without a fee."

But our would-be student, not being entirely satisfied with the advice given, calls in the evening on "that modest fellow with the absurd title, D. D. S." The dentist receives the young man politely, and learning his mission invites him to be seated, and to excuse him for a few moments while he finishes a letter he is writing to a former classmate.

While the dentist is finishing the letter the young man glances around the waiting-room, and his eye resting on the bookcase, he rises from his seat to examine the kind of books a dentist reads, and discovers to his surprise that many of the books on the shelves are exact copies of the identical text-books that his friend, the M. D., has in his bookcase. It puzzles him a little, but the letter is finished, and, the dentist returning, the two enter into conversation.

*Young Man*—Why I see you use the same kind of books that Dr. Pompous, our family physician, uses.

*Dentist*—Yes, to a very large extent, but not altogether, as there are some medical books which, while they are essential to a physician, would be comparatively useless to a dentist, as they do not come within the range of his practice; however, we try to keep posted, and therefore read everything which we think will aid us in a more thorough understanding of our specialty; but of course as the fundamental principles which underlie both general and special practice are the same, we must of necessity use the same general text-books, viz.: those on chemistry, anatomy, physiology, pathology, therapeutics, etc., and that will account for the books you see on my shelves. But in addition to the general text-books we have quite a list that treat more extensively of our specialty, with which we must also become familiar. All these are used in our dental colleges.

*Young Man*—Then you think a medical college is not the place for a young man to go in order to study dental surgery?

*Dentist*—Most certainly not. Not but that there are many things taught in a medical college that are absolutely necessary for a dentist to know in order to qualify him for the practice of his profession. These essential things are taught in our dental colleges, but are so arranged as to combine both theory and practice, thus making it far easier for the student and much safer for the public, from the fact that when a graduate in dentistry goes forth from the college in which he received his instruction he does so as a practitioner; young, it is true, with much to learn, nevertheless a practitioner, well grounded in the principles and practice of his profession. Not so with the medical graduate. He leaves his Alma Mater with ideas, facts and theories all commingled, having had no opportunity to separate the wheat from the chaff, and therefore all the practical part of his profession he has yet to learn outside of the college walls, and that too after he has been duly accredited as fully competent to practice his profession. No, my dear sir, a medical college is not the place to study dentistry, for as a rule the professors know absolutely nothing about the dental organs, except what they may have gleaned in a general sort of way. They are not to blame by any means, for as you grow older you will learn that this world, within and without the colleges, is made up largely of two-talent men, who can hold only so much at a time.

*Young Man*—But would it not be best if one had the time and money to first graduate in general medicine and then take up the specialty?

*Dentist*—There are some very worthy dentists who think so, but I think not, for the reason that it would seem to me like taking a long, circuitous, uncertain route through the woods, not knowing where you will come out, rather than to travel over a shorter, more direct and fairly beaten path by which to reach the same point. Besides, no man can serve two masters, and you would have to leave one to serve the other, for by dividing the service you could be faithful to neither.

*Young Man*—Why so?

*Dentist*—Because, as I said, this world is run, so to speak, by the two-talent men, and pardon me in assuming you to be one of such. You have neither the brain-power to retain the knowledge, or the physical ability to perform the labor necessary; to use another simile, it is impossible to put a quart of water into a pint cup. You can only fill the cup, and as most of us dentists hold only about a pint we must therefore keep ourselves filled with that kind of knowledge that will do us the most good and make us the most useful in the profession we have chosen. By first taking the medical course you will have gained but little; your mechanical ability will have to be developed, your eye educated, your mind taught to conceive and to fully realize and to appreciate how such a simple thing as a human tooth, when in an abnormal condition, may be instrumental in causing bodily disease, and sometimes even death.

*Young Man*—But I understand your dental colleges are not all models of perfection?

*Dentist*—Not by any means, and let me assure you there are none who feel more keenly and appreciate the fact so much as the high minded, right thinking dentist, who would it were otherwise, and there are many such in the ranks of our profession. Rome was not built in a day, and it will take time to fully perfect and round out our young profession to its ultimate proportions, for like our country and its form of government, the dental profession was left an orphan with none to take it in. Yes, literally, out in the cold. But its members have struggled on, fighting with the weapons of their warfare until, like our country's flag, emblem of liberty, the American dental ensign of D. D. S., is known and (when not carried by pirates) respected throughout the civilized world.

*Young Man*—But have you no M. D.'s who practice dentistry exclusively?

*Dentist*—Oh, yes, quite a large number, and I assure you some of them are large hearted men, to whom the profession owes a debt of gratitude, for they have helped to formulate and arrange our course of studies and in many other ways have materially assisted us in the progress we have made as dentists. We have some M. D.'s amongst us, however, who are so wedded to their Alma Maters and their old ways of teaching that it warps their judgment in matters pertaining to dental schools. But we have amongst us another class of M. D.'s who, while they are good men and excellent dentists and who doubtless means well, cannot look at matters dental except through the too strongly focused old medical school glasses, which distorts their vision and thus unfits them for safe advisers in dental school education. But it is said that 'revolutions never go backward,' and the dental college being of American origin will stand as such, from time to time improving its methods, until all that seems crude and unfinished shall have disappeared, and dental surgery shall have become symmetrical in all its proportions as a branch of the Healing Art. * * * So, my dear sir, if my advice is worth anything, I would say first, that if you wish to become a dentist in something more than name, you must avoid the professional pirate, whether he sails a large ship or a small one, for we have both kinds. Secondly, place yourself with a respectable, well-informed dental surgeon, who will direct you in your preliminary studies and properly prepare you for a dental college. Thirdly, don't bother yourself about the mystical title of M. D., for after you have honorably graduated as a dental surgeon you will know why it is that in this country, at least, the once honored title of M. D. is largely a thing of the past. And when you are a dental surgeon, if you be a true man and would do unto others as you would they should do unto you, you will then seek, in general medicine, in the arts and sciences, or wherever else it may be found, what you will feel you most need—more light and more knowledge.

